# Outbreak and epidemic of Getah virus infection in swine by virulence-enhanced GIII variant in Henan, central China in 2024

**DOI:** 10.1080/21505594.2025.2530661

**Published:** 2025-07-13

**Authors:** Yawei Sun, Han Yang, Yingshuo Zhang, Ruiwu Liu, Leiyin Li, Mengmeng Shi, Tao Liu, Jimei Du, Zhanda Guo, Li Zhao, Yongtao Li, Linyang Yu, Lu Chen

**Affiliations:** a College of Veterinary Medicine, Henan Agricultural University, Zhengzhou, Henan Province, China; b Department of Biosafety, United Graduate School of Chinese Modern Agriculture, Zhengzhou, Henan Province, China; c College of Animal Science and Technology, Xinyang Agriculture and Forestry University, Xinyang, Henan Province, China; d Henan Dahe Agricultural and Animal Husbandry Technology Company, Zhengzhou, Henan Province, China

**Keywords:** Getah virus GIII variant, mosquito-borne virus, genetic characteristics, outbreak and epidemic, increased virulence

## Abstract

The Getah virus (GETV), an emerging mosquito-borne virus, poses a threat to many animal species and probably even humans. Between late July and mid-September, concentrated outbreaks of GETV on pig farms have occurred in multiple regions across Henan, and showed epidemic characteristics. To investigate the genetic characteristics and virulence changes of GETV strains responsible for the GETV epidemic in Henan, a total of 27 GETV strains were isolated from 31 commercial pig farms located in 21 counties. Phylogenetic and sequence alignment analysis results showed that 22 of the isolates formed a distinct cluster within the phylogenetic tree, and the strains in this cluster exhibited 3 amino acid mutations in the non-structural protein nsP3 along with an amino acid mutation in the structural protein E2. Piglet pathogenicity experiments demonstrated that the isolate could induce the characteristic clinical signs of GETV infection in piglets, resulting in 100% mortality. Comparative analysis of pathogenicity in mice showed that the pathogenicity of the GETV GIII variant was significantly enhanced compared with the early isolate. This study is the first to investigate in detail the large-scale, concentrated outbreak and epidemic of GETV infection in pig farms in Henan and confirms that there is a trend of increasing virulence of the GIII variant responsible for this outbreak. The data in this study will contribute to the understanding of the molecular epidemiological characteristics and pathogenicity dynamics of GETV.

## Introduction

Getah virus is a positive single strand RNA virus, that belongs to the *Alphavirus* genus of the *Togaviridae* family, with a gene length of 11.7kb [1]. The genome comprises two non-coding regions (5’UTR and 3’ UTR), two open reading frames (ORF1 and ORF2), and a 26S RNA binding region situated between ORF1 and ORF2 [2]. ORF1 occupies the initial two-thirds of the genome and encodes four non-structural proteins (nsP1, nsP2, nsP3, and nsP4), among which nsP3 is associated with viral pathogenicity [3]. Previous studies have demonstrated that the absence of nsP3 in alphaviruses reduces their virulence, thereby establishing them as potential candidates for vaccine development [4,5]. The nsP3 protein is composed of a highly conserved N-terminal macro domain (MD), a central zinc-binding domain (ZBD), and a C-terminal hypervariable domain (HVD) [6]. Situated in the terminal third of the genome, ORF2 encodes five structural proteins (C, E3, E2, 6K, and E1), of which the E2 protein is closely associated with viral adsorption and elicitation of host immune responses [7,8].

In 1955, the GETV strain was initially isolated from Malaysian mosquitoes and has subsequently exhibited rapid dissemination across Eurasia and the Pan-Pacific, thereby exemplifying its extensive geographical distribution [9]. GETV is classified into four distinct genotypes, namely GI, GII, GIII, and GIV, based on the E2 gene sequence [10]. Among these genotypes, GIII group is currently the most prevalent and poses a significant threat to animal health. The GETV exhibits a remarkably broad host range, encompassing mosquitoes, horses, pigs, cattle [11], foxes [12], and red pandas [13]. Moreover, recent studies have shown the potential of farmed fur-bearing raccoon dog as hosts for GETV infection [14]. The clinical signs of horses infected with GETV included fever, rash, and hind limb oedema [15]. Piglets show clinical signs including diarrhoea, tremor, and hind limb paralysis when infected with GETV [16]. Sows infected with the GETV often experience a high rate of abortion and produce weak litters [17,18]. In addition, initial serological investigations have demonstrated the presence of antibodies against GETV in human serum, suggesting a possible correlation between GETV infection and febrile illnesses in humans [19].

Since the first isolation of the GETV strain in China in 1964, most provinces in China have been affected by GETV [20,21], but the outbreaks of GETV occurred sporadically [18,22,23]. In the current study, we reported a concentrated outbreak and epidemic of GETV on commercial pig farms in Henan Province, central China, between late July and mid-September 2024. we isolated 27 strains from disease samples collected from pig farms affected by the GETV in Henan provinces. The dominant strains responsible for the GETV epidemic were the GIII variant, which was more virulent than earlier isolate. These findings will contribute to the understanding of the current molecular epidemiological characteristics and pathogenicity dynamics of GETV, and will inform its prevention and control.

## Materials and methods

### Cells and viruses

BHK-21 (ATCC, CCL-10) and PK-15 (ATCC, CCL-33) cells were maintained in Dulbecco’s modified Eagle’s medium (DMEM, Hyclone) supplemented with 10% heat-inactivated foetal bovine serum (FBS, Gibco), penicillin (100 IU/mL), and streptomycin (100 µg/mL) at 37°C in a humidified 5% CO_2_ atmosphere.

The GETV strain HNJZ-S1, the first GETV isolate from pigs in China [24], was isolated in 2017 and preserved in our laboratory.

### Sample collection and detection

Between late July to September in 2024, samples of suspected GETV infection were collected from commercial pig farms in Henan and Shanxi, China. The tissue or blood samples were frozen immediately after collection and delivered to Henan Agricultural University in frozen-sealed condition. According to the manufacturer’s instructions, viral DNA/RNA was extracted from the samples using the Tiangen DNA/RNA co-extraction kit (Cat. RT411), and cDNA synthesis was performed using a Vazyme HiScript® II Q RT Supermix (Vazyme, Nanjing, China) (Cat. R323–01). GETV and other viruses (pseudorabies virus [PRV], porcine parvovirus [PPV], porcine circovirus type 2 [PCV2], porcine deltacoronavirus [PDCoV], porcine epidemic diarrhoea virus [PEDV], Japanese encephalitis virus [JEV], porcine reproductive and respiratory syndrome virus [PRRSV], and porcine transmissible gastroenteritis virus [TGEV]) were tested for by PCR or RT-PCR. The virus detection primers used in this study are listed in Supplementary Table S1. The visualization of maps of Henan Province, China, was done using ArcGIS 10.8, and the labelling of the locations of GETV-positive pig farms was done using Adobe Illustrator 2020.

### Isolation, identification, and growth characteristics

For virus isolation, homogenate supernatants of PCR-positive samples were filtered using a 0.22-μm filter, and inoculated into BHK-21 cells. The supernatants and cells with obvious cytopathic effects (CPE) were harvested following three freeze-thaw cycles. The isolates were propagated on BHK-21 cells followed by five passages, and then three rounds of plaque purification.

The isolated strains were identified using RT-PCR, and the positive samples were named based on their area of origin. Further identification was performed by transmission electron microscopy as described previously [25].

The HNzk-XH1 and HNzmd-XP1 isolates were inoculated onto BHK-21 and PK-15 cells in 6-well plates at an MOI of 0.01. After incubation for 2 h, the cells were washed twice with PBS and then cultured in DMEM containing 2% FBS. The cell culture was collected at 12, 24, 36, 48, 60, and 72 hours post infection (hpi). After three cycles of freezing and thawing, cell debris was eliminated by centrifugation at 8000 r/min for 5 minutes, after which the supernatant was collected and inoculated onto BHK-21 cells. The 50% tissue culture infectious dose (TCID_50_) of GETV was calculated using the Reed-Muench method. Each experiment was performed in triplicate to determine the mean titre for multistep growth curves.

For viral plaque assays, BHK-21 cells grown to 90% confluence in 6-well plates were infected with the HNzk-XH1 and HNzmd-XP1 strains at 100 TCID_50_ for 2 h. The cells were then overlaid with a mixture of DMEM containing 1% low-melting agarose (Lonza, Cat. No: 50302) and 2% FBS. At 24 hpi, the infected cells were fixed with 4% paraformaldehyde at 4°C for 30 mins, and then the medium was carefully removed and the cells were washed twice with PBS. The cells were stained with 1% crystal violet staining solution for 30 mins and then washed twice with PBS to observe the visible plaques.

### Sequencing, phylogenetic and sequence alignment analysis

As described above, cDNAs were obtained from cells infected with various isolates. All PCR amplification primers used for sequencing are listed in Supplementary Table S2. The E2 gene was amplified and then cloned into pMD-18T (Takara, Cat. No: 6011) for sequencing. For whole genome sequencing, the complete genome of the isolates was divided into 12 segments for amplification and then cloned into pMD-18T for sequencing. Positive plasmids were sent to Sunya Biotechnology Co., Ltd. (Zhengzhou, China) for bidirectional sequencing by Sanger sequencing. Sequences were assembled using the BioEdit software and were deposited in GenBank.

The phylogenetic analysis of GETV strains was made based on E2 or the complete genome, available sequences of the GETV strains were obtained from the National Center for Biotechnology Information (NCBI) (Supplementary Table S3). The tree was constructed using the maximum likelihood (ML) method with 1,000 bootstrap replicates in MEGA 7.0 (www.megasoftware.net) and then was modified by iTOL (https://itol.embl.de/). We used organism silhouettes from PhyloPic (www.phylopic.org) to further annotate the evolutionary tree in Adobe Illustrator 2020.

The nucleotide sequences of E2 and the whole genome were edited and aligned using BioEdit software and then translated into amino acids from each gene and compared using DNASTAR Lasergene.v7.1 (DNASTAR, Inc., Madison, WI, USA) The sequence alignment results were drawn using ESpript 3.0 (https://espript.ibcp.fr/ESPript/ESPript/).

### Pathogenicity in piglet

Eight one-day-old colostrum-deprived piglets were purchased from a local GETV/PRRSV/PRV/*Mycoplasma hyopneumoniae*-negative pig farm. Upon arrival, the piglets were monitored for 24 hours, during which no abnormal signs were observed. Subsequently, the piglets were randomly assigned into two groups using a computer-based random order generator and housed in a room at a constant temperature of 30°C at Henan Agricultural University Pig Diseases Institute. Piglets in challenge group (*n* = 4) received an intramuscular injection of 10^7^ TCID_50_ of the HNzk-XH1 strain. Piglets in the negative control group (*n* = 4) were injected with DMEM. Each piglet was considered an experimental unit. Clinical signs were evaluated every 12 hours and clinical scores were based on the following criteria: 0 for healthy, 1 for depression, 2 for diarrhoea, 3 for hind limb paralysis, and 4 for death. Clinical signs were assessed independently by two authors and disagreements were resolved by a third author. The sum of the points was recorded as the clinical score, with a maximum value of 4. Piglets without any clinical disease were scored 0. The third author who performed randomization and the second author who analysed the data were unaware of the group assignment, while the rearing investigators (the first, fourth, fifth and sixth authors) were aware of the group assignments.

At 2 days post-challenge (dpc), 2 mL blood was collected from piglets into the blood collection tubes containing ethylenediaminetetraacetic acid (EDTA, Solarbio, Cat. E8040). Piglets that died were necropsied immediately. At 8 dpc, the piglets in the negative control group were euthanized. The liver, spleen, lung, kidney, small intestine and brain samples were collected, and the viral loads in the tissues were determined by RT-qPCR.

### Virulence test in suckling mice

140-day-old SPF pregnant ICR mice were purchased from Liaoning Changsheng Biotechnology Co., Ltd. (Liaoning, China). Pregnant mice (about 18 days gestation) were kept alone and gave birth 2–4 days later. The Suckling mice were evenly divided, and each mother fed 12 pups. Pregnant and lactating mice were fed the appropriate food and sterile water according to the recommendations of the mouse supplier. The IVC system is suitable for housing SPF-grade mice. Each pregnant mouse was housed separately in an individually ventilated cage (IVC, FENGSHI TECH, ZJ-4, China) at the College of Veterinary Medicine of Henan Agricultural University throughout the study period.

For the determination of median lethal dose (LD50), two-day-old SPF ICR mice (*n* = 78) were randomly divided into twelve challenge groups (6 in each group) and one negative control group (*n* = 6) using a computer-based random order generator. Suckling mice in challenge groups received a subcutaneous injection of 10^1^ to 10^6^ TCID_50_ of the HNzk-XH1 or HNZJ-S1 strain. Suckling mice in the negative control group were injected with DMEM. Each suckling mouse was considered an experimental unit. In addition, the third author who performed randomization and the first author who analysed the data were unaware of the group assignment, and the rearing investigator (the second author) was aware of the group assignments. The suckling mice were observed for death every 12 hours and weighed every 24 hours. The LD50 of the virus strains was calculated by the Reed-Muench method.

### Viral RNA load determination

Tissue samples collected for the piglet experiment in this study were weighed. A volume of PBS corresponding to the sample’s mass was added, the samples were homogenized and freeze-thawed three times to release the virus, and the homogenates were centrifuged at 8000 r/min for 5 min. According to the manufacturer’s instructions, the viral RNA was extracted from 200 μL supernatants using the Tiangen RNA extraction kit (Cat. DP419), and cDNA synthesis was performed using the HiScript QRT SuperMix for qPCR (+gDNA wiper) Kit (Vazyme, Cat. R323–01).

SYBR green qPCR was performed using primers (GETV-nsP1-qF: AGC ATT TTC GCA TCT GGC TAC, GETV-nsP1-qR: TCT GGG TCT TCC GCA CTT TT) targeting the nsP1 gene of GETV [26]. The qRT-PCR reaction volume was 20 μL, including 2× Universal SYBR Green Fast qPCR Mix (ABclone, Cat. RK21203), forward and 0.4 μM reverse primers, template DNA, and sterile water. The reaction conditions were denaturation at 95°C for 3 min, followed by 40 cycles of 95°C for 5 s and 60°C for 34 s, with relative quantification analysis based on the cycle threshold method.

### Statistical analysis

The data were analysed by GraphPad Prism 9.0 (GraphPad Software Inc. La Jolla, CA, USA) and expressed by mean ± standard deviation (SD).

## Results

### GETV sample collection and detection

**Figure 1.**
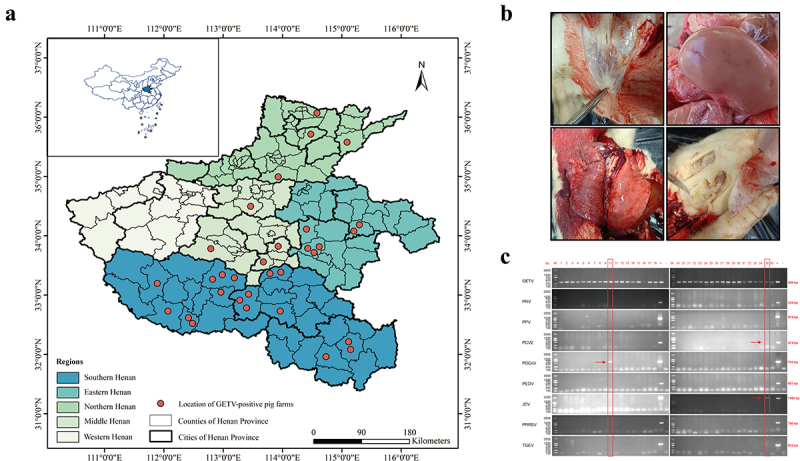
GETV infection epidemic information. (a). Location of GETV-positive pig farms in Henan province. The base layer of this modified map originated from National Earth System Science data center, National Science & Technology infrastructure of China (http://www.Geodata.cn). (b) Gross tissue lesions of infected piglets in this outbreak. Top left: lymphadenopathy; Top right: kidney spot bleeding; bottom left: lung haemorrhage; bottom right: subcutaneous oedema. (c) PCR identification of GETV and other viruses (PRV, PPV, PCV2, PDCoV, PEDV, JEV, PRRSV, and TGEV) in tissue samples.

In July 2024, a disease outbreak with high mortality occurred among piglets aged 3 to 10 days in a swine facility located in Zhumadian City, Henan Province, resulting in an incidence of 30% and mortality of 80%. Our lab confirmed that the outbreak was attributed to a single strain of GETV. Subsequently, suspected outbreaks of GETV emerged in numerous pig farms located in Henan province, and we collected clinical samples from commercial pig farms. The statistical results showed that GETV epidemics were observed in 12 of the 18 cities, and 21 of the 157 counties (Figure 1(a)). The statistical data on the epidemic situation of GETV showed that there was an outbreak in July, 15 outbreaks in August, and 16 outbreaks in September (Table 1). In piglets, GETV infection was characterized by severe diarrhoea, ataxia, and hind limb weakness. The gross pathology at necropsy showed lymph node enlargement with haemorrhage, pulmonary haemorrhage, scattered surface bleeding spots, and subcutaneous oedema (Figure 1(b)). The detection of other porcine-origin viruses in the collected samples showed that out of the 36 GETV positive samples from pig farms, only two samples exhibited co-infection of GETV with PDCoV, and GETV with PCV2 and JEV, while the remaining outbreaks were solely attributed to GETV (Figure 1(c)).

**Table 1.** Sample information collected in this study.

No.	Farm No.	Time	Isolate name	Area	Pig stage
1	1	31-Jul-2024	/	Dengzhou; Nanyang; Henan	piglet
2	2	6-Aug-2024	/	Neixiang; Nanyang; Henan	piglet
3	3	8-Aug-2024	/	Sheqi; Nanyang; Henan	piglet
4	4	17-Aug-2024	/	Xinye; Nanyang; Henan	piglet
5	5	21-Aug-2024	HNny-XY	Xinye; Nanyang; Henan	piglet
6	6	5-Aug-2024	HNzmd-BY1	Biyang; Zhumadian; Henan	piglet
7	7	22-Aug-2024	/	Biyang; Zhumadian; Henan	piglet
8	8	6-Sep-2024	/	Biyang; Zhumadian; Henan	piglet
9	9	18-Aug-2024	HNzmd-XP1	Xiping; Zhumadian; Henan	piglet
10		22-Aug-2024	HNzmd-XP2		
11	10	19-Aug-2024	HNzmd-XP3	Xiping; Zhumadian;Henan	piglet
12		3-Sep-2024	HNzmd-XP4		piglet
13	11	1-Sep-2024	HNzmd-QS	Queshan; Zhumadian; Henan	piglet
14	12	16-Aug-2024	HNlh-LY	Linying; Luohe; Henan	piglet
15	13	17-Aug-2024	HNzk-XH1	Xihua; Zhoukou; Henan	piglet
16	14	31-Aug-2024	HNzk-FG	Fugou; Zhoukou; Henan	piglet
17	15	18-Aug-2024	HNxx-YY	Yuanyang; Xinxiang; Henan	piglet
18	16	26-Aug-2024	HNzz-XM	Xinmi; Zhengzhou; Henan	piglet
19	17	30-Aug-2024	HNsq-ZC	Zhecheng; Shangqiu; Henan	piglet
20	18	3-Sep-2024	/	Zhecheng; Shangqiu; Henan	piglet
21	19	1-Sep-2024	HNpds-LS	Lushan; Pingdingshan; Henan	piglet
22	20	1-Sep-2024	HNpy-PY	Puyang; Puyang; Henan	Sow
23	21	6-Sep-2024	HNxy-HC1	Huangchuan; Xinyang; Henan	piglet
24	22	6-Sep-2024	HNxy-HC2	Huangchuan; Xinyang; Henan	piglet
25	23	8-Sep-2024	HNhb-XX	Xunxian; Hebi; Henan	piglet
26	24	19-Sep-2024	HNzk-XH2	Xihua; Zhoukou; Henan	piglet
27	25	19-Sep-2024	HNzk-XH3	Xihua; Zhoukou; Henan	piglet
28	26	17-Sep-2024	HNxy-GS	Guangshan; Xinyang; Henan	piglet
29	27	18-Sep-2024	HNny-FC1	Fangcheng; Nanyang; Henan	piglet
30		18-Sep-2024	HNny-FC2	Fangcheng; Nanyang; Henan	Sow
31	28	18-Sep-2024	HNny-FC3	Fangcheng; Nanyang; Henan	piglet
32		18-Sep-2024	HNny-FC4	Fangcheng; Nanyang; Henan	piglet
33	29	20-Sep-2024	HNny-FC5	Fangcheng; Nanyang; Henan	piglet
34	30	18-Sep-2024	HNlh-WY	Wuyang; Luohe; Henan	Sow
35	31	19-Sep-2024	/	Anyang; Anyang; Henan	piglet
36	32	6-Sep-2024	SXxz-FS	Fanshi; Xinzhou; Shanxi	Sow

### Isolation, identification, and virus titer determination of GETV

**Figure 2.**
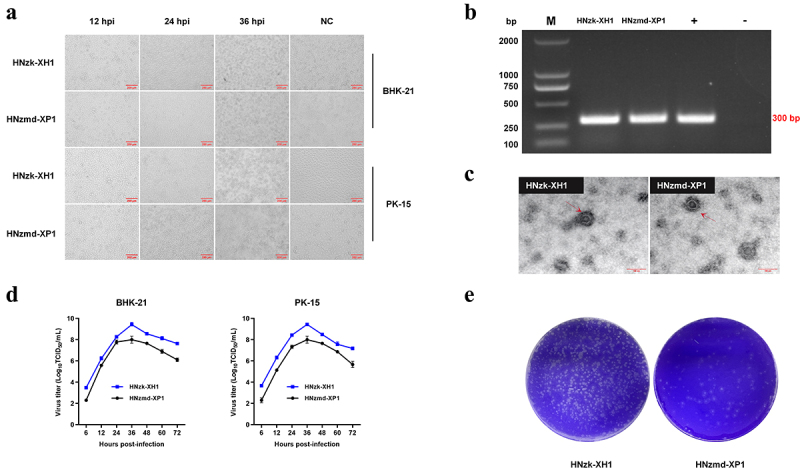
Isolation and characterization of GETV. (a). BHK-21 and PK15 cells were infected with HNzk-XH1 or HNzmd-XP1 (MOI = 0.01). Cytopathic were observed at 12, 24, and 36 hpi. (B) GETV isolates were identified by RT-PCR. (c) Electron microscopic examination of morphology of GETV particles. BHK-21 cells were infected with HNzk-XH1 and HNzmd-XP1, and the precipitated viruses from the supernatants were processed for electron microscopy. (d) Growth of HNzk-XH1 or HNzmd-XP1 in BHK-21 and PK-15 cells. Viral titres were determined for samples at 12, 24, 36, 48, 60, and 72 hpi in BHK-21 cells. Data are expressed as the mean ± standard deviation (SD) of viral titres (log10 TCID_50_ per 1 ml) derived from three independent experiments. (E) plaque morphology of GETV on BHK-21 cells. Monolayers of BHK-21 cells in six-well plates were infected with HNzk-XH1 and HNzmd-XP1 (100 TCID_50_). The cell monolayers were overlaid with 1% agarose and stained with crystal violet at 24 hpi.

A total of 28 strains of GETV were successfully isolated from BHK-21 lines in 36 collected positive samples from 36 piglets or sows. The isolated HNzk-XH1 and HNzmd-XP1 strains were used for virus isolation and identification. The results showed that BHK-21 or PK15 cells inoculated with purified HNzk-XH1 or HNzmd-XP1 showed a significant CPE in the form of shrinking, rounding and detachment of cells at 48 hours post-infection (hpi) compared to that of the control (Figure 2(a)). The causative agent of CPE was identified as the GETV by RT-PCR (Figure 2(b)) and electron microscopy (Figure 2(c)). The viral proliferation curve indicated both GETV strains had similar growth kinetics, with the peak viral titre reached at 36 hours post-infection, followed by a subsequent decline (Figure 2(d)). The virus plaque assay revealed that HNzk-XH1 and HNzmd-XP1 produced plaques of similar size but varied in plaque counts (Figure 2(e)).

### Phylogenetic and sequencing analysis

The complete genome (GenBank accession numbers: PQ658739-PQ658750) and E2 gene sequence (GenBank accession numbers: PQ658751-PQ658766) obtained in this study have been uploaded to the GenBank database. The whole genome identity analysis showed that the nucleotide identity among the isolates was 98.4%-100.0%, and the amino acid identity was 99.3%-100.0%. The nucleotide identity between the isolates in this study and the reference strains of GI, GII, GIV and GIII was 94.8%-95.0%, 96.9%-97.0%, 95.6%-97.3%, and 96.9%-99.8%, and the amino acid identity were 98.5%-98.8%, 98.9%-99.1%, 98.3%-99.5%, and 98.7%-100.0%, respectively (Supplementary Table S4).

**Figure 3.**
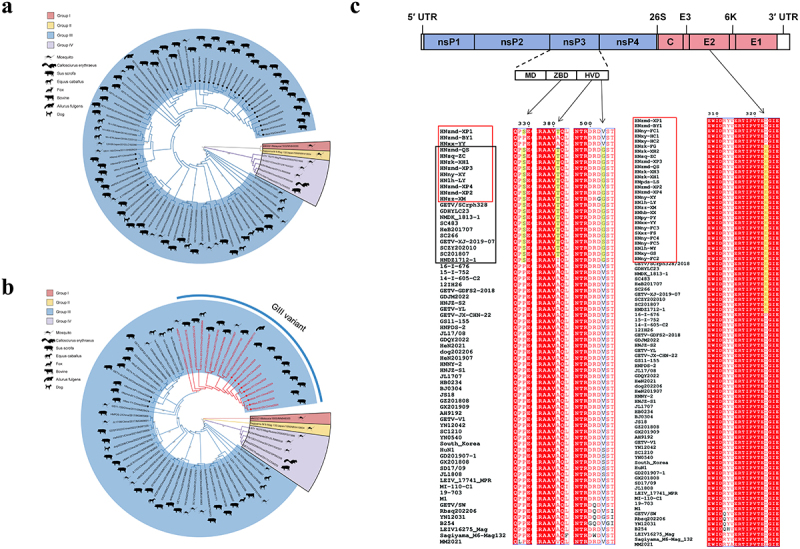
Phylogenetic analysis and amino acid sequence alignment of the isolates in this study. Phylogenetic analysis of isolates based on E2 (a) and complete genome (b). The phylogenetic tree were generated using the maximum likelihood (ML) method implemented in the program MEGA 7.0. Bootstrap values are represented as a percentage based on 1,000 replicates. The strains obtained in this study are represented by a closed circle. The GIII variants are indicated in red font. (c) Amino acid sequence alignment based on complete genome. The GIII variants are in the black frame; the isolates in this study are in the red frame; common amino acid mutations of the GIII variant that differ from other GETV GIII strains are highlighted in yellow.

Phylogenetic trees were constructed based on the E2 gene of 28 isolates and the whole genomes of 12 selected isolates, respectively. The results indicated that all isolates belonged to the GIII group and exhibited a distant genetic relationship with other groups (Figure 3(a,b)). Additionally, most of the isolates were in the same branch as the GDHYLC23 variant (Figure 3(b)).

The analysis of amino acid sequence alignment revealed that the GETV variants have four unique amino acid mutations in the nsP3 and E2 proteins (Figure 3(c)). Specifically, the nsP3 protein has three amino acid mutations: one in the ZBD (P329S), and two in the HVD (A381T and V503G). Additionally, the E2 protein has an amino acid mutation at position 323, which changes aspartic acid to glutamic acid (D323E). Based on these findings, we determined this branch strains as GIII variant.

### Pathogenicity of GETV HNzk-XH1 strain in piglets

**Figure 4.**
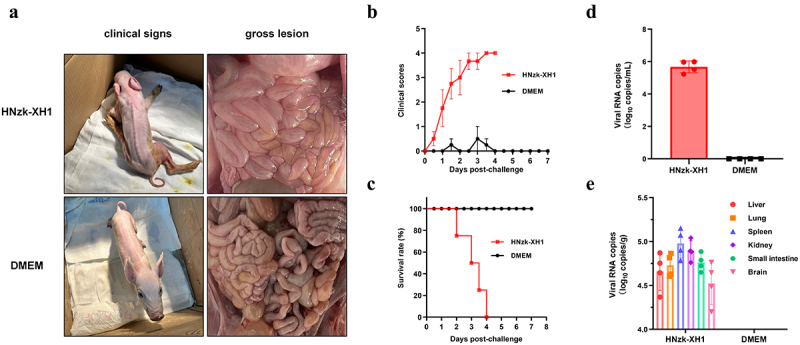
Pathogenicity in piglets. (a) Clinical signs and general autopsy of infected piglets. (b) clinical scores of infected piglets. Depression = 1, diarrhea = 2, Hind limb paralysis = 3, death = 4, and health = 0. (c) survival rate of infected piglets. (d) viral RNA levels in whole blood of infected piglets on day 2 post challenge. (e) viral RNA levels in different organs of piglets that died post challenge or euthanized.

Considering the high mortality associated with the GETV outbreak in commercial pig farms and the high viral titre observed in GETV isolated from BHK-21 and PK-15 cells, we selected the HNzk-XH1 strain, which exhibits a high viral titre, to evaluate its pathogenicity in piglets. Piglets in the challenge group were intramuscularly injected with 10^7^ TCID_50_ of the HNzk-XH1 strain, while the control group received intramuscular injections of DMEM. At 24 h post-challenge, the piglets in the challenge group began to show signs of diarrhoea, hind limb paralysis was observed at 1.5 dpc, one piglet died at 2 dpc, and all piglets died at 4 dpc, resulting in a mortality of 100% (Figure 4(c)). The clinical signs were scored (Figure 4(b)). The severity of clinical signs in the HNzk-XH1 group was significantly higher compared to the control group. The piglets that died in the HNzk-XH1 group were dissected immediately, and the piglets in the control group were dissected at 8 dpc. The observation shows that the small intestine of piglets in the HNzk-XH1 group exhibited a significantly reduced thickness and was characterized by the presence of yellow, watery contents. In contrast, the small intestine of piglets in the control group displayed normal morphology (Figure 4(a)). The blood samples of piglets collected at 2 dpc were analysed using RT-qPCR. The results revealed a copy number of nearly 10^6^/ml for the GETV nsP1 gene in the challenge group, while no nsP1 gene was tested in the control group (Figure 4(d)). The liver, lung, kidney, spleen, small intestine, and brain of dissected piglets were tested by RT-qPCR. All examined organs of piglets from the group injected with the HNzk-XH1 variant had high viral load, whereas no GETV RNA was detected in the negative control group (Figure 4(e)).

### Pathogenicity alterations of HNzk-XH1 variant in suckling mice

**Figure 5.**
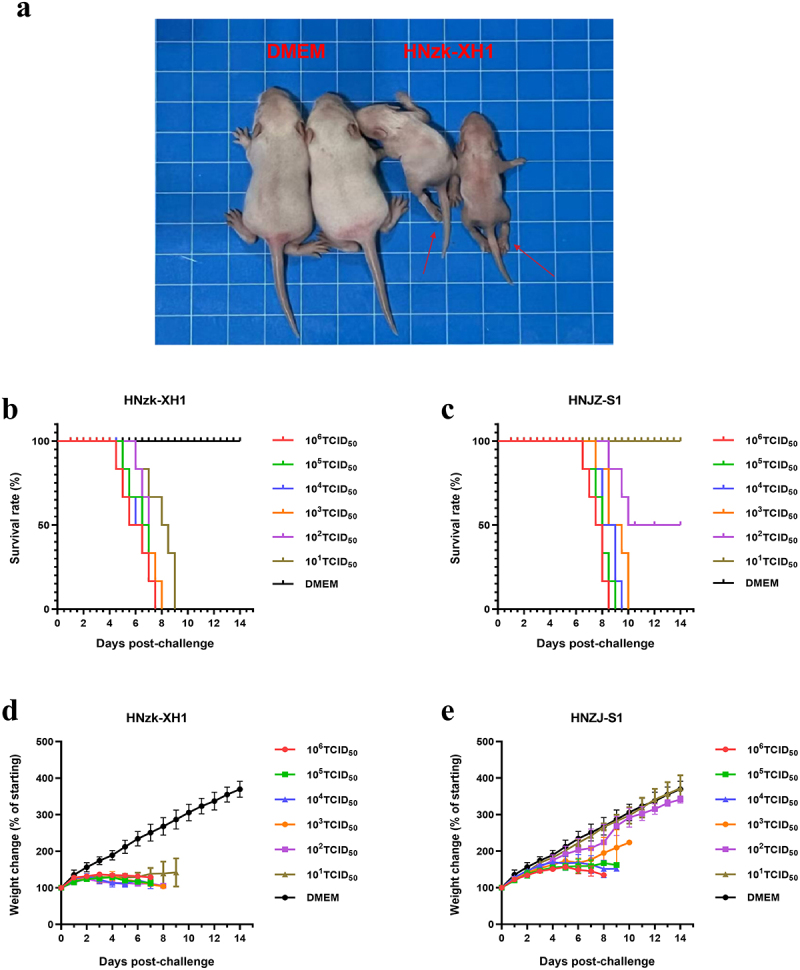
Comparison of pathogenicity between HNzk-XH1 and HNZJ-S1. (a) Clinical signs of infected suckling mice. (b) Survival rate of suckling mice at different infective doses of the HNzk-XH1 strain. (c) Survival rate of suckling mice at different infective doses of the HNZJ-S1 strain. (d) Weight change of suckling mice at different infective doses of the HNzk-XH1 strain. (e) Weight change of suckling mice at different infective doses of the HNZJ-S1 strain.

The present research demonstrates that suckling mice serve as optimal models for investigating the virulence of GETV [7,27,28]. Therefore, we selected the HNzk-XH1 variant to challenge suckling mice at a dose of 10^5^ TCID_50_. The experimental results showed that the challenge group exhibited hind limb paralysis and growth arrest (Figure 5(a)). Subsequently, we conducted comparative pathogenicity studies on suckling mice using the HNzk-XH1 strain and HNZJ-S1 strain isolated by our laboratory in 2017 at different doses. The results showed that suckling mice in the HNzk-XH1 group, challenged with the highest dose (10^6^ TCID_50_) appeared to die at 4.5 dpc, and all suckling mice had died by 7.5 dpc. In contrast, the HNzk-XH1 group injected with the lowest dose (10^1^ TCID_50_) appeared to die at 6 dpc and all suckling mice had died by 9 dpc (Figure 5(b)). However, the results from suckling mice challenged with the HNZJ-S1 strain showed that suckling mice injected with the highest dose (10^6^ TCID_50_) appeared to die at 6.5 dpc, and all suckling mice had died by 8.5 dpc (Figure 5(c)). Suckling mice injected with a dose of 10^2^ TCID_50_ began to die at 8.5 dpi, yielding a final mortality rate of 50%, while suckling mice injected with a dose of 10^1^ TCID_50_ exhibited 100% survival. All suckling mice with challenge HNzk-XH1 variant exhibited impaired growth compared with the control group (Figure 5(d)). However, suckling mice challenged with HNJZ-S1 strain that survived the challenge showed no alteration in the growth rate of body weight compared with the control group (Figure 5(e)).

## Discussion

The spread of emerging and re-emerging vector-borne infectious diseases has been gradually expanding. GETV is a mosquito-borne pathogen that poses a potential threat to public health.

In recent years, there have been successive reports of outbreaks of GETV infection in pig farms across various provinces in China, but all of them occurred in single-farm spots [22,29]. However, between late July and mid-September 2024, we observed a concentrated outbreak and epidemic of GETV infection in Henan Province. We confirmed that this outbreak, through differential diagnosis, virus isolation and identification, and pathogenicity in piglets, was caused by GETV. Our investigation alone found 31 GETV outbreaks across 21 counties in Henan Province (Figure 1(a)), and the actual spread of the epidemic may be even wider. This widespread outbreak has caused significant economic losses to the pig industry.

Henan Province, a major pig farming region in China and home to numerous large pig enterprises, has been recognized as a potential centre for GETV transmission [20]. In 2017, our lab successfully isolated the first porcine-derived GETV HNZJ-S1 strain in China [24]. The HNZJ-S2 strain and other strains were subsequently isolated [30], including the GETV-V1 strain from a commercially available PRRSV vaccine [31]. Epidemiological studies indicate that Nanyang, an important large-scale pig farming city in southwestern Henan Province, was the earliest and most outbreak-prone city in the current round of epidemic (Table 1). Smallholder pig farmers purchasing pregnant sows and weaned piglets from large-scale farms for rearing is one of the main farming models after the African swine fever outbreak in 2018. Therefore, the practice of trading pigs with infected farms may have contributed to the rapid spread of the current epidemic.

The occurrence and development of arboviral diseases are driven by the “virus-arbovector-host” triad [32,33]. In this study, a virulence test conducted in suckling mice revealed that the virulence of the GETV variants had increased during the recent outbreak, which may have contributed to the epidemic (Figure 5). However, this study’s pathogenicity comparison focused solely on virus-host interactions, limiting its ability to accurately represent changes in the transmission and pathogenicity of variants during the epidemic. The pathogenicity comparison experiment in this study only involved virus-host interactions, which made it difficult to accurately reflect the changes in transmission and pathogenicity of the mutant strains during this epidemic. Changes in the infectivity of variants to mosquitoes and alterations in the pathogenicity of mosquitoes infected by variants to their hosts may also have contributed to the current round of epidemics, which requires further research. Amino acid mutations possibly increase the transmissibility and pathogenicity of viruses, contributing to the rapid spread and frequent outbreaks of arboviral diseases [34]. In this study, phylogenetic analysis based on the E2 gene and whole genome sequencing indicated that all isolates belonged to GIII group, with 23 out of 28 isolates clustering on the same branch (Figure 3A). Notably, recent GETV strains isolated from infected piglets and mosquitoes also belong to this branch, suggesting that these strains may be emerging as dominant [18,29,35]. Additionally, the results of the GETV evolutionary dynamics study showed the variant branch (representative strain: NMDK1813–1, SC483) evolved the fastest in the GIII group [36].

The virulence of GETV is associated with several virulence factors, including the E2 and nsP3 proteins. The E2 is a receptor-binding protein that associates with viral adsorption [7,37,38]. The ZBD in the middle of nsP3 is closely associated with viral replication [39]. The HVD at the C-terminus of nsP3 is highly variable and mainly mediates interactions with the host [3,40,41]. Amino acid sequence alignment analysis revealed that this branch strain possesses a unique amino acid mutation at position 323 of the E2 protein (D323E) and three unique mutations in the nsP3 protein, located in the ZBD (P329S) and HVD (A381T and V503G) (Figure 3(c)). Based on these findings, we determined this branch strain as GIII variant. Codon Glu323 of the E2 protein is one of the positive selection sites for the adaptive evolution of GETV [36]. Phosphorylation sites on alphavirus nsP3 are associated with viral replication [42]. GETV variants introduced additional phosphorylation sites with amino acid mutations (P329S and A381T) on the nsP3 protein. The effect of these mutations on GETV needs to be further investigated. Previous studies have demonstrated that adaptive mutations in the nsP1 and capsid proteins of the Zika virus enhance its infectivity in hosts and mosquitoes [43]. Similarly, a single amino acid mutation in the envelope protein of CHIKV enhances viral replication in Aedes albopictus, thereby increasing transmission by this mosquito vector [44]. In the case of GETV, a single amino acid mutation at position 253 of the E2 protein can significantly affect its virulence [7]. Whether the four amino acid mutations identified in the variants are responsible for increased infectivity in hosts and mosquitoes, and consequently for the outbreaks and epidemic, warrants further exploration through reverse genetic technique.

The transmission of arboviruses is significantly influenced by external conditions, including climate, topography, and temperature [45]. Research indicates that global warming is facilitating a notable expansion of mosquito-borne infectious disease zones in China, particularly from south to north, and is also contributing to an increase in the frequency and intensity of epidemics [46]. According to geographic location, Henan Province is divided into five geographic regions (eastern Henan, western Henan, southern Henan, northern Henan, and central Henan). The southern Henan lies within the subtropical zone, whereas the other regions fall within the temperate zone. In this study, 17 out of 31 outbreaks were identified in southern Henan (Figure 1(a)), suggesting that the outbreak distribution is influenced by climate, although the limited number of samples may also play a role.

Mosquito activity peaks in the summer, however, the earliest outbreak in this study was detected in late July. This timing implies that GETV may have been circulating between livestock which do not exhibit obvious signs and mosquitoes, before the epidemic. Pigs serve as amplifying hosts, and a gradual increase in GETV infection among mosquitoes is anticipated following the current outbreak, potentially leading to a broader and more hazardous subsequent outbreak, with an increased risk of spillover to other animals and even humans. It is crucial to recognize that GETV primarily causes severe disease in pups and pregnant animals. Therefore, relying solely on serological investigations of the general population may obscure the true risk to humans, particularly if studies neglect key groups such as pregnant women, newborns, and aborted foetuses. Additionally, the absence of a GETV vaccine in China precludes immunization as a preventive measure against future outbreaks. An inactivated combination vaccine against JEV and GETV is commercially available in Japan for use in racehorses [47]. However, this type of vaccine seems to be less effective [48]. Live attenuated vaccines provide long-lasting protection but pose safety concerns. Subunit vaccines are safe, easy to produce on a large scale, and widely used for controlling livestock diseases. Therefore, developing a subunit vaccine against GETV is an attractive option [49]. Continuous monitoring of the epidemiological and molecular characteristics of GETV in pigs and mosquitoes, along with timely warnings to farms, are essential strategies to prevent further epidemic expansion and spillover. Furthermore, the development of a GETV vaccine should be prioritized.

In summary, we observed a concentrated outbreak and widespread epidemic of GETV infection in pig farms in Henan, China, from late July to mid-September 2024. The GETV variants causing this outbreak were identified as belonging to the same independent branch of the GIII group and exhibit four amino acid mutations in the nsP3 and E2 proteins, distinct from other strains. Additionally, the GETV variants showed a trend of increased virulence compared with the early isolate.

## Supplementary Material

Supplementary Table 1.docx

Supplementary Table 2.docx

Supplementary Table 4.docx

Supplementary Table 3.docx

## Data Availability

The sequencing data (accession numbers: PQ658739-PQ658766) that support the findings of this study are openly available in GenBank. The data that support the findings of this study are directly available in ScienceDB (http://www.doi.org/10.57760/sciencedb.20137).

## References

[1] Hu T, Zheng Y, Zhang Y, et al. Identification of a novel Getah virus by Virus-Discovery-cDNA random amplified polymorphic DNA (RAPD). BMC Microbiol. 2012;12(1):305. doi: 10.1186/1471-2180-12-305 23268691 PMC3547691

[2] Zhai Y, Wang H, Sun X, et al. Complete sequence characterization of isolates of Getah virus (genus Alphavirus, family Togaviridae) from China. J Gen Virol. 2008;89(6):1446–13. doi: 10.1099/vir.0.83607-0 18474561

[3] Qi X, Zhao R, Yao X, et al. Getah virus Nsp3 binds G3BP to block formation of bona fide stress granules. Int J Biol Macromol. 2024;279:135274. doi: 10.1016/j.ijbiomac.2024.135274 39226976

[4] Hallengärd D, Kakoulidou M, Lulla A, et al. Novel attenuated Chikungunya vaccine candidates elicit protective immunity in C57BL/6 mice. J Virol. 2014;88(5):2858–2866. doi: 10.1128/JVI.03453-13 24371047 PMC3958085

[5] Schmidt C, Schnierle B. Chikungunya Vaccine Candidates: Current Landscape and Future Prospects. Drug Des Devel Ther. 2022;16:3663–3673. doi: 10.2147/DDDT.S366112 PMC9580835 36277603

[6] Götte B, Liu L, McInerney G. The Enigmatic Alphavirus Non-Structural Protein 3 (nsP3) Revealing Its Secrets at Last. Viruses. 2018;10(3):105. doi: 10.3390/v10030105 29495654 PMC5869498

[7] Wang N, Zhai X, Li X, et al. Attenuation of getah virus by a single amino acid substitution at residue 253 of the E2 protein that might be part of a new heparan sulfate binding site on alphaviruses. J Virol. 2022;96(6):e01751–01721. doi: 10.1128/jvi.01751-21 34986000 PMC8941864

[8] Voss JE, Vaney MC, Duquerroy S, et al. Glycoprotein organization of Chikungunya virus particles revealed by X-ray crystallography. Nature. 2010;468(7324):709–712. doi: 10.1038/nature09555 21124458

[9] Shi N, Zhu X, Qiu X, et al. Origin, genetic diversity, adaptive evolution and transmission dynamics of Getah virus. Transbound Emerg Dis. 2022;69(4):e1037–e1050. doi: 10.1111/tbed.14395 34812572

[10] Li YY, Liu H, Fu SH, et al. From discovery to spread: The evolution and phylogeny of Getah virus. Infect Genet Evol. 2017;55:48–55. doi: 10.1016/j.meegid.2017.08.016 28827175

[11] Liu H, Zhang X, Li LX, et al. First isolation and characterization of Getah virus from cattle in northeastern China. BMC Vet Res. 2019;15(1):320. doi: 10.1186/s12917-019-2061-z 31488162 PMC6729113

[12] Shi N, Li LX, Lu RG, et al. Highly Pathogenic Swine Getah Virus in Blue Foxes, Eastern China, 2017. Emerg Infect Dis. 2019;25(6):1252–1254. doi: 10.3201/eid2506.181983 31107236 PMC6537705

[13] Zhao M, Yue C, Yang Z, et al. Viral metagenomics unveiled extensive communications of viruses within giant pandas and their associated organisms in the same ecosystem. Sci Total Environ. 2022;820:153317. doi: 10.1016/j.scitotenv.2022.153317 35066043

[14] Zhao J, Wan W, Yu K, et al. Farmed fur animals harbour viruses with zoonotic spillover potential. Nature. 2024;634(8032):228–233. doi: 10.1038/s41586-024-07901-3 39232170 PMC11741233

[15] Fukunaga Y, Kumanomido T, Kamada M. Getah Virus as an Equine Pathogen. Vet Clin North Am Equine Pract. 2000;16(3):605–617. doi: 10.1016/s0749-0739(17)30099-8 11219353

[16] Yago K, Hagiwara S, Kawamura H, et al. A fatal case in newborn piglets with Getah virus infection: isolation of the virus. Nihon Juigaku Zasshi. 1987;49:989–994. doi: 10.1292/jvms1939.49.1003 2828741

[17] Kumanomido T, Wada R, Kanemaru T, et al. Clinical and virological observations on swine experimentally infected with Getah virus. Vet Microbiol. 1988;16(3):295–301. doi: 10.1016/0378-1135(88)90033-8 2836997

[18] Wu Y, Gao X, Kuang Z, et al. Isolation and pathogenicity of a highly virulent group III porcine Getah virus in China. Front Cell Infect Microbiol. 2024;14:1494654. doi: 10.3389/fcimb.2024.1494654 39483122 PMC11524988

[19] Li XD, Qiu FX, Yang H, et al. Isolation of Getah virus from mosquitos collected on Hainan Island, China, and results of a serosurvey. Southeast Asian J Trop Med Public Health. 1992;23(4):730–734. 1338481

[20] Zhao J, Dellicour S, Yan Z, et al. Early Genomic surveillance and phylogeographic analysis of getah virus, a reemerging arbovirus, in livestock in china. J Virol. 2023;97(1):e01091–01022. doi: 10.1128/jvi.01091-22 36475767 PMC9888209

[21] Lu G, Chen R, Shao R, et al. Getah virus: An increasing threat in China. J Infect. 2020;80(3):350–371. doi: 10.1016/j.jinf.2019.11.016 31790706

[22] Yang T, Li R, Hu Y, et al. An outbreak of Getah virus infection among pigs in China, 2017. Transbound Emerg Dis. 2018;65(3):632–637. doi: 10.1111/tbed.12867 29575687

[23] Lu G, Ou J, Ji J, et al. Emergence of Getah Virus Infection in Horse With Fever in China, 2018. Front Microbiol. 2019;10:1416. doi: 10.3389/fmicb.2019.01416 31281304 PMC6596439

[24] Zhou F, Cui DD, Wang AJ, et al. Isolation and identification of the first getah virus (GETV) strain HNJZ-S1 from clinically suspected PRRS case of pig herd in Henan Province, China. Chin J Virol. 2018;34(1):59–66. doi: 10.13242/j.cnki.bingduxuebao.003299

[25] Ren T, Mo Q, Wang Y, et al. Emergence and phylogenetic analysis of a getah virus isolated in southern china. Front Vet Sci. 2020;7:552517. doi: 10.3389/fvets.2020.552517 33344520 PMC7744783

[26] Cao X, Qiu X, Shi N, et al. Establishment of a reverse transcription real-time quantitative PCR method for Getah virus detection and its application for epidemiological investigation in Shandong, China. Front Microbiol. 2022;13:1009610. doi: 10.3389/fmicb.2022.1009610 36212868 PMC9538719

[27] Wang A, Zhou F, Liu C, et al. Structure of infective Getah virus at 2.8 Å resolution determined by cryo-electron microscopy. Cell Discov. 2022;8(1):12. doi: 10.1038/s41421-022-00374-6 35149682 PMC8832435

[28] Zhai X, Li X, Veit M, et al. LDLR is used as a cell entry receptor by multiple alphaviruses. Nat Commun. 2024;15(1):622. doi: 10.1038/s41467-024-44872-5 38245515 PMC10799924

[29] Chu PP, Guo H, Zhou X, et al. Emergence of a novel GIII Getah virus variant in pigs in Guangdong, China, 2023. Microbiol Spectr. 2024;12(8):e00483–00424. doi: 10.1128/spectrum.00483-24 38916356 PMC11302130

[30] Wang A, Zhou F, Chang H, et al. Molecular detection, isolation and identification of the porcine Getah virus from pig herds in Four Provinces, China. Chin J Virol. 2018;34:522–532. doi: 10.13242/j.cnki.bingduxuebao.003398

[31] Zhou F, Wang A, Chen L, et al. Isolation and phylogenetic analysis of Getah virus from a commercial modified live vaccine against porcine reproductive and respiratory syndrome virus. Mol Cell Probe. 2020;53:101650. doi: 10.1016/j.mcp.2020.101650 32781023

[32] Cox J, Mota J, Sukupolvi-Petty S, et al. Mosquito Bite Delivery of Dengue Virus Enhances Immunogenicity and Pathogenesis in Humanized Mice. J Virol. 2012;86(14):7637–7649. doi: 10.1128/jvi.00534-12 22573866 PMC3416288

[33] Schneider BS, Higgs S. The enhancement of arbovirus transmission and disease by mosquito saliva is associated with modulation of the host immune response. Trans R Soc Trop Med Hyg. 2008;102:400–408. doi: 10.1016/j.trstmh.2008.01.024 18342898 PMC2561286

[34] Yuan L, Huang XY, Liu ZY, et al. A single mutation in the prM protein of Zika virus contributes to fetal microcephaly. Science. 2017;358(6365):933–936. doi: 10.1126/science.aam7120 28971967

[35] Wu Q, Sun D, Zaman W, et al. Detection and evolutionary characterization of arboviruses in mosquitoes and biting midges on Hainan Island, China, 2019–2023. PLOS Negl Trop Dis. 2024;18(10):e0012642. doi: 10.1371/journal.pntd.0012642 39480881 PMC11556698

[36] Shen J, Liu S, Liu S, et al. Genomic surveillance and evolution of Getah virus. Virus Evol. 2025;11(1):veaf007. doi: 10.1093/ve/veaf007 39989716 PMC11844246

[37] Jose J, Snyder JE, Kuhn RJ. A structural and functional perspective of alphavirus replication and assembly. Future Microbiol. 2009;4(7):837–856. doi: 10.2217/fmb.09.59 19722838 PMC2762864

[38] Silva LA, Khomandiak S, Ashbrook AW, et al. A Single-Amino-Acid Polymorphism in Chikungunya Virus E2 Glycoprotein Influences Glycosaminoglycan Utilization. J Virol. 2014;88(5):2385–2397. doi: 10.1128/JVI.03116-13 24371059 PMC3958064

[39] Wang YF, Sawicki SG, Sawicki DL. Alphavirus nsP3 functions to form replication complexes transcribing negative-strand RNA. J Virol. 1994;68(10):6466–6475. doi: 10.1128/jvi.68.10.6466-6475.1994 8083984 PMC237067

[40] Kim D, Reynaud J, Rasalouskaya A, et al. New World and Old World Alphaviruses Have Evolved to Exploit Different Components of Stress Granules, FXR and G3BP Proteins, for Assembly of Viral Replication Complexes. PLOS Pathog. 2016;12(8):e1005810. doi: 10.1371/journal.ppat.1005810 27509095 PMC4980055

[41] Panas MD, Varjak M, Lulla A, et al. Sequestration of G3BP coupled with efficient translation inhibits stress granules in Semliki Forest virus infection. Mol Biol Cell. 2012;23(24):4701–4712. doi: 10.1091/mbc.e12-08-0619 23087212 PMC3521679

[42] Teppor M, Žusinaite E, Merits A, et al. Phosphorylation sites in the hypervariable domain in chikungunya virus nsP3 are crucial for viral replication. J Virol. 2021;95(9):0. doi: 10.1128/JVI.02276-20 PMC8104096 33568506

[43] Liu Y, Liu J, Du S, et al. Evolutionary enhancement of Zika virus infectivity in Aedes aegypti mosquitoes. Nature. 2017;545(7655):482–486. doi: 10.1038/nature22365 28514450 PMC5885636

[44] Tsetsarkin KA, Vanlandingham DL, McGee CE, et al. A single mutation in chikungunya virus affects vector specificity and epidemic potential. PLOS Pathog. 2007;3(12):e201. doi: 10.1371/journal.ppat.0030201 18069894 PMC2134949

[45] Waldock J, Chandra NL, Lelieveld J, et al. The role of environmental variables on Aedes albopictus biology and chikungunya epidemiology. Pathog Glob Health. 2013;107(5):224–241. doi: 10.1179/2047773213Y.0000000100 23916332 PMC4001452

[46] Khan M, Pedersen M, Zhu M, et al. Dengue transmission under future climate and human population changes in mainland China. Appl Math Model. 2023;114:785–798. doi: 10.1016/j.apm.2022.10.027

[47] Bannai H, Nemoto M, Ochi A, et al. Epizootiological Investigation of Getah Virus Infection among Racehorses in Japan in 2014. J Clin Microbiol. 2015;53(7):2286–2291. doi: 10.1128/jcm.00550-15 25972425 PMC4473224

[48] Nemoto M, Bannai H, Tsujimura K, et al. Getah Virus Infection among Racehorses, Japan, 2014. Emerg Infect Dis. 2015;21(5):883. doi: 10.3201/eid2105.141975 25898181 PMC4412242

[49] Miao Q, Nguyen W, Zhu J, et al. A getah virus-like-particle vaccine provides complete protection from viremia and arthritis in wild-type mice. Vaccine. 2024;42(25):126136. doi: 10.1016/j.vaccine.2024.07.037 39004524

