# Supplementary material for: Outbreak and epidemic of Getah virus infection in swine by virulence-enhanced GIII variant in Henan, central China in 2024
Source: Virulence. 2025 Jul 13;16(1):2530661. doi: 10.1080/21505594.2025.2530661 (PMC12269664; doi:10.1080/21505594.2025.2530661)
Supplement: Supplementary Table 1.docx [file KVIR_A_2530661_SM2269.docx]

**Supplementary Table 1.** Primers used to detect GETV or other viruses.

| **Primer** | **Sequence(5′–3′)** | **Length of amplification (bp)** |
| --- | --- | --- |
| TGEV-F | GGAGTTGTCTGGGTTGCCAA | 816 |
| TGEV-R | GTTCGTTACCTCATCAATCAT |  |
| PRRSV-F | CAACACCCAGGCGACTTCAGAAATG | 796 |
| PRRSV-R | CCAAGTCAGCATGTCAACCCTATC |  |
| JEV-F | TTTAATTGTCTGGGAATGGGC | 1500 |
| JEV-R | AGCATGCACATTGGTCGCTAA |  |
| PPV-F | ATGAGTGAAAATGTGGAAC | 974 |
| PPV-R | GTGTAGCTATTATTAATTG |  |
| PRV-F | CGGCTTCCACTCGCAGCTCTTC | 339 |
| PRV-R | GTGTGCACCTCCTCGCCGAAG |  |
| PCV2-F | CCAGCAAAAAGAATGGAAGAAG | 474 |
| PCV2-R | ACGGGGTCTGATTGCTGGTAATC |  |
| PDCoV-F | GTGAGGCTCGCTCGGCTGAATG | 702 |
| PDCoV-R | GCAGAACATCATCAGGTTGAG |  |
| PEDV-F | GACAAACCCCTTTCTAAGGTAC | 651 |
| PEDV-R | CTTAAGCTTGTCAGGGTTTTCG |  |
| GETV-F | ACCGAAGAAGCCGAAGAA | 300 |
| GETV-R | GCACTCRAGGTCATACTTG |  |
