# Supplementary material for: Outbreak and epidemic of Getah virus infection in swine by virulence-enhanced GIII variant in Henan, central China in 2024
Source: Virulence. 2025 Jul 13;16(1):2530661. doi: 10.1080/21505594.2025.2530661 (PMC12269664; doi:10.1080/21505594.2025.2530661)
Supplement: Supplementary Table 2.docx [file KVIR_A_2530661_SM2268.docx]

**Supplementary Table 2.** GETV amplification primer used for sequencing

| **Primer** | **Site in genome** | **Sequence(5′**-**3′)** | **Amplify region** | **Length of amplification (bp)** |
| --- | --- | --- | --- | --- |
| GETV-F1 | 1-21 | ATGGCGGACGTGTGACATCAC | 5′ UTR-nsP1 | 1206 |
| GETV-R1 | 1206-1183 | GTTTGTGTTTCTTTGCGTCCTACC |  |  |
| GETV-F2 | 983-1006 | ACGCAGTTACCCATCACGCAGAGG | nsP1-nsP2 | 1185 |
| GETV-R2 | 2167-2144 | CGTGGAAAGGTGGATTGATTAGGT |  |  |
| GETV-F3 | 2009-2031 | GGCCAGCCTTGAATACCGACGAG | nsP2 | 1101 |
| GETV-R3 | 3109-3086 | CTTTGGCCTTGTTTTGGAATGGAT |  |  |
| GETV-F4 | 2920-2937 | TTGACCCGAACTGAAAAC | nsP2 | 972 |
| GETV-R4 | 3891-3872 | CACCACCATCTCGCTGACTC |  |  |
| GETV-F5 | 3794-3815 | AGATGCTGGGAGGGGATTCACT | nsP2-nsP3 | 1082 |
| GETV-R5 | 4875-4859 | CGCGTACCGACATAGAC |  |  |
| GETV-F6 | 4734-4757 | GCCAAGACTCCAAGACGYTAACGA | nsP3 | 939 |
| GETV-R6 | 5672-5649 | GGGCCAGTGTCAGACGAAAAGATA |  |  |
| GETV-F7 | 5507-5526 | CGTGGGAGCCGGAAGACCTA | nsP3-nsP4 | 816 |
| GETV-R7 | 6322-6302 | CTACGTTRAACACCGCAGAGT |  |  |
| GETV-F8 | 6202-6220 | GCAGTACCTTCCCCTTTTC | nsP4 | 1098 |
| GETV-R8 | 7299-7276 | TTTTAGTGCCCTTCTTCTGTCTTC |  |  |
| GETV-F9 | 7102-7122 | TGGGTCAACATGGAAGTAAAG | nsP4-C protein | 1022 |
| GETV-R9 | 8123-8101 | ACCGTGATGCCAATTGTAGTGCC |  |  |
| GETV-F10 | 7933-7956 | TAGTCGGGGATAAAGTGATGAAGC | C protein-E2 | 1121 |
| GETV-R10 | 9053-9030 | GTCTGGGATGTCCGGTGGGGTATG |  |  |
| GETV-F11 | 8923-8943 | CCCCAGTAGGCAGAGAAAAAT | E2-E1 | 1346 |
| GETV-R11 | 10268-10248 | CTCAGTGTCGCAGAAGCAGTA |  |  |
| GETV-F12 | 10085-10102 | GGAACCCACRCTAAACTT | E1-3′ UTR | 1605 |
| GETV-R12 | 11689–11665 | GTAAAATATTAAAAAAACAAATTAG |  |  |
| GETV-E2 | 8523-8544 | AGTGTGACGGAACACTTCAATG | E2 | 1266 |
| GETV-E2 | 9788-9767 | GGCATGCGCTCGTGGTGCGCAG |  |  |
