# Supplementary material for: Outbreak and epidemic of Getah virus infection in swine by virulence-enhanced GIII variant in Henan, central China in 2024
Source: Virulence. 2025 Jul 13;16(1):2530661. doi: 10.1080/21505594.2025.2530661 (PMC12269664; doi:10.1080/21505594.2025.2530661)
Supplement: Supplementary Table 4.docx [file KVIR_A_2530661_SM2267.docx]

**Supplementary Table 4.** Nucleotide and amino acid sequence and identity analyses of the GETV isolates and reference strains

| **Comparison** | **Nucleotide (%)** | | | **Amino acid (%)** | | |
| --- | --- | --- | --- | --- | --- | --- |
|  | **ORF1** | **ORF2** | **Complete genome** | **ORF1** | **ORF2** | **Complete genome** |
| Comparison among the isolates | 98.4-100.0 | 98.4-100.0 | 98.4-100.0 | 99.1-100.0 | 99.5-100.0 | 99.3-100.0 |
| Compared with other GETV GIII strains | 96.8-99.8 | 96.9-99.8 | 96.9-99.8 | 98.1-100.0 | 98.6-100.0 | 98.7-100.0 |
| Compared with GETV GIV strains | 95.7-97.3 | 95.1-97.3 | 95.6-97.3 | 98.1-99.2 | 98.1-99.5 | 98.3-99.5 |
| Compared with GETV GII strains | 97.0-97.1 | 96.5-96.8 | 96.9-97.0 | 98.6-98.9 | 98.8-99.1 | 98.9-99.1 |
| Compared with GETV GI strains | 94.8-95.2 | 94.5-94.8 | 94.8-95.0 | 98.1-98.3 | 98.1-98.4 | 98.5-98.8 |
