# Supplementary material for: Outbreak and epidemic of Getah virus infection in swine by virulence-enhanced GIII variant in Henan, central China in 2024
Source: Virulence. 2025 Jul 13;16(1):2530661. doi: 10.1080/21505594.2025.2530661 (PMC12269664; doi:10.1080/21505594.2025.2530661)
Supplement: Supplementary Table 3.docx [file KVIR_A_2530661_SM2266.docx]

**Supplementary Table 3.** Information of the GETV isolates and reference strains in this study

| **Strain** | **Country** | **Date** | **Host** | **Genbank No** |
| --- | --- | --- | --- | --- |
| **HNzmd-XP1** | **China** | **2024** | **Pig** | **PQ658739^Complete genome^** |
| **HNzmd-XP2** | **China** | **2024** | **Pig** | **PQ658740^Complete genome^** |
| **HNzmd-XP3** | **China** | **2024** | **Pig** | **PQ658741^Complete genome^** |
| **HNzk-XH1** | **China** | **2024** | **Pig** | **PQ658742^Complete genome^** |
| **HNny-XY** | **China** | **2024** | **Pig** | **PQ658743^Complete genome^** |
| **HNlh-LY** | **China** | **2024** | **Pig** | **PQ658744^Complete genome^** |
| **HNxx-YY** | **China** | **2024** | **Pig** | **PQ658745^Complete genome^** |
| **HNzmd-QS** | **China** | **2024** | **Pig** | **PQ658746^Complete genome^** |
| **HNzz-XM** | **China** | **2024** | **Pig** | **PQ658747^Complete genome^** |
| **HNzmd-XP4** | **China** | **2024** | **Pig** | **PQ658748^Complete genome^** |
| **HNzmd-BY1** | **China** | **2024** | **Pig** | **PQ658749^Complete genome^** |
| **HNsq-ZC** | **China** | **2024** | **Pig** | **PQ658750^Complete genome^** |
| **HNny-FC1** | **China** | **2024** | **Pig** | **PQ658751^E2^** |
| **HNxy-HC1** | **China** | **2024** | **Pig** | **PQ658752^E2^** |
| **HNxy-HC2** | **China** | **2024** | **Pig** | **PQ658753^E2^** |
| **HNzk-FG** | **China** | **2024** | **Pig** | **PQ658754^E2^** |
| **HNzk-XH2** | **China** | **2024** | **Pig** | **PQ658755^E2^** |
| **HNzk-XH3** | **China** | **2024** | **Pig** | **PQ658756^E2^** |
| **HNpds-LS** | **China** | **2024** | **Pig** | **PQ658757^E2^** |
| **HNhb-XX** | **China** | **2024** | **Pig** | **PQ658758^E2^** |
| **HNpy-PY** | **China** | **2024** | **Pig** | **PQ658759^E2^** |
| **HNny-FC3** | **China** | **2024** | **Pig** | **PQ658760^E2^** |
| **SXxz-FS** | **China** | **2024** | **Pig** | **PQ658761^E2^** |
| **HNny-FC4** | **China** | **2024** | **Pig** | **PQ658762^E2^** |
| **HNny-FC5** | **China** | **2024** | **Pig** | **PQ658763^E2^** |
| **HNlh-WY** | **China** | **2024** | **Pig** | **PQ658764^E2^** |
| **HNxy-GS** | **China** | **2024** | **Pig** | **PQ658765^E2^** |
| **HNny-FC2** | **China** | **2024** | **Pig** | **PQ658766^E2^** |
| GDHYLC23 | China | 2023 | Pig | OR487192 |
| SCZY202010 | China | 2020 | Pig | OP004828 |
| SC201807 | China | 2018 | Pig | MK693225 |
| SC266 | China | 2018 | Pig | MN478487 |
| SC483 | China | 2018 | Pig | MN478486 |
| HeB201707 | China | 2017 | Sus scrofa | MZ736793 |
| HNDZ1712-1 | China | 2017 | Culex tritaeniorhynchus | ON828425 |
| NMDK 1813-1 | China | 2018 | Mosquito | MW512827 |
| GETV/SCrph328/2018 | China | 2018 | Ailurus fulgens | MZ357111 |
| GETV-XJ-2019-07 | China | 2019 | Equus caballus | MZ388464 |
| GDJM2022 | China | 2022 | Pig | ON843770 |
| GETV-JX-CHN-22 | China | 2022 | Pig | OQ968487 |
| GDQY2022 | China | 2022 | Pig | ON987235 |

**Continued Supplementary Table 3.** Information of the GETV isolates and reference strains in this study

| **Strain** | **Country** | **Date** | **Host** | **Genbank No** |
| --- | --- | --- | --- | --- |
| GETV-YL | China | 2021 | Pig | OL352731 |
| GETV-GDFS2-2018 | China | 2018 | Pig | MT086508 |
| JS18 | China | 2018 | Pig | MT210319 |
| GX201808 | China | 2018 | Pig | MT269657 |
| HuN1 | China | 2017 | Pig | MF741771 |
| AH9192 | China | 2017 | Pig | MG865965 |
| HNPDS-2 | China | 2017 | Pig | MG865969 |
| GETV-V1 | China | 2016 | Pig | KY399029 |
| HNNY-2 | China | 2016 | Pig | MG865967 |
| HNJZ-S2 | China | 2015 | Pig | KY363863 |
| HNJZ-S1 | China | 2011 | Pig | KY363862 |
| South Korea | South Korea | 2004 | Pig | AY702913 |
| HeN2021 | China | 2021 | Sus scrofa | MZ736800 |
| GX201909 | China | 2019 | Sus scrofa | MZ736795 |
| HeN201907 | China | 2019 | Sus scrofa | MZ736792 |
| GD201907-1 | China | 2019 | Sus scrofa | MZ736798 |
| BJ0304 | China | 2003 | Sus scrofa | OM363683 |
| dog202206 | China | 2022 | Dog | OP593309 |
| JL1808 | China | 2018 | Cattle | MH722256 |
| SD17/09 | China | 2017 | Fox | MH106780 |
| GZ201808 | China | 2018 | Equus caballus | MK487997 |
| 19-703 | Japan | 2019 | Equus caballus | LC710657 |
| 16-I-676 | Japan | 2016 | Equus caballus | LC223132 |
| 15-I-752 | Japan | 2015 | Equus caballus | LC212972 |
| 14-I-605-C2 | Japan | 2014 | Equus caballus | LC079089 |
| MI-110-C1 | Japan | 1978 | Equus caballus | LC079086 |
| JL17/08 | China | 2017 | Mosquito | MG869691 |
| JL1707 | China | 2017 | Mosquito | MH722255 |
| YN12042 | China | 2012 | Culex tritaeniorhynchus | KY450683 |
| 12IH26 | Japan | 2012 | Culex tritaeniorhynchus | LC152056 |
| GS11-155 | China | 2011 | Culex tritaeniorhynchus | ON828424 |
| HB0234 | China | 2002 | Culex tritaeniorhynchus | EU015062 |
| SC1210 | China | 2012 | Armigeres subalbatus | LC107870 |
| YN0540 | China | 2005 | Armigeres subalbatus | EU015063 |
| M1 | China | 2008 | Culex sp. | EU015061 |
| LEIV 17741 MPR | Mongolia | 2000 | Culex sp. | EF631999 |
| Rbsq202206 | China | 2022 | Callosciurus erythraeus Pallas | OP593308 |
| GETV/SW | Thailand | 2017 | Sus scrofa | LC534253 |
| YN12031 | China | 2012 | Armigeres subalbatus | KY434327 |
| B254 | Malaysia | 2012 | Culex fuscocephalus | LR990838 |
| LEIV 16275 Mag | Russia | 2000 | Aedes sp. | EF631998 |

**Continued Supplementary Table 3.** Information of the GETV isolates and reference strains in this study

| **Strain** | **Country** | **Date** | **Host** | **Genbank No** |
| --- | --- | --- | --- | --- |
| Sagiyama M 6-Mag 132 | Japan | 1956 | Mosquito | MW410934 |
| MM2021 | Malaysia | 1955 | Culex gelidus | MN849355 |

**Note:** Black and bold are the isolates in this study
